# Associations between psychosocial factors and work ability in a Tunisian electricity and gas company

**DOI:** 10.1192/j.eurpsy.2023.666

**Published:** 2023-07-19

**Authors:** N. Rmadi, N. Kammoun, A. Hrairi, N. Kotti, R. Masmoudi, K. Jmal Hammami, M. L. Masmoudi, J. Masmoudi, M. Hajjaji

**Affiliations:** ^1^Department Of Occupational Medicine, HEDI CHAKER hospital, University of Sfax; ^2^ Tunisian Ocupational Health and Safety Institute; ^3^Psychiatry A Department, HEDI CHAKER hospital, University of Sfax, SFAX, Tunisia

## Abstract

**Introduction:**

Work ability can be influenced by numerous factors, particularly psychosocial ones. These latter can be individual psychosocial factors but also psychosocial factors at the workplace.

**Objectives:**

This study aimed to explore psychosocial determinants of work ability among workers in a Tunisian electricity and gas company.

**Methods:**

We conducted a cross-sectional survey among 83 male workers in a Tunisian electricity and gas company. We used a self-administered questionnaire that included socio-demographic profile, psychosocial factors assessment through the Job content questionnaire (JCQ) and General Health Questionnaire (GHQ-12), and Work Ability Index (WAI) questionnaire. Data were analysed using SPSS software. We used the student’s test to compare means between two groups.

**Results:**

The mean WAI score among workers in the studied electricity and gas company was 8.96 (SD=1.37). At the time of the survey, one person out of 3 among the participants suffered from a psychological distress (37.3% with a GHQ-12 score ≥ 3). These Workers had a weaker work ability compared to those with not (p=0.033). We found also that having low social support and passive jobs were associated with low work ability (p=0.003 and p=0.005 respectively).

**Conclusions:**

Most personal and occupational psychosocial factors had significant associations with WAI in the studied company. Thus, enhancing the psychosocial environment in the workplace can promote work ability in such occupations.

**Disclosure of Interest:**

None Declared

